# Efficient Minimum Flow Decomposition via Integer Linear Programming

**DOI:** 10.1089/cmb.2022.0257

**Published:** 2022-11-08

**Authors:** Fernando H.C. Dias, Lucia Williams, Brendan Mumey, Alexandru I. Tomescu

**Affiliations:** ^1^ Department of Computer Science, University of Helsinki, Helsinki, Finland.; ^2^ School of Computing, Montana State University, Bozeman, Montana, USA.

**Keywords:** flow decomposition, integer linear programming, multiassembly and RNA assembly, network flow

## Abstract

Minimum flow decomposition (MFD) is an NP-hard problem asking to decompose a network flow into a minimum set of paths (together with associated weights). Variants of it are powerful models in multiassembly problems in Bioinformatics, such as RNA assembly. Owing to its hardness, practical multiassembly tools either use heuristics or solve simpler, polynomial time-solvable versions of the problem, which may yield solutions that are not minimal or do not perfectly decompose the flow. Here, we provide the first fast and exact solver for MFD on acyclic flow networks, based on Integer Linear Programming (ILP). Key to our approach is an encoding of *all* the exponentially many solution paths using only a *quadratic* number of variables. We also extend our ILP formulation to many practical variants, such as incorporating longer or paired-end reads, or minimizing flow errors. On both simulated and real-flow splicing graphs, our approach solves *any* instance in <13 seconds. We hope that our formulations can lie at the core of future practical RNA assembly tools. Our implementations are freely available on Github.

## 1. INTRODUCTION

Flow decomposition (FD), the problem of decomposing a network flow into a set of weighted source-to-sink paths that perfectly explains the flow values on the edges, is a classical and well-studied concept in computer science. For example, it is a standard result that any flow in a directed acyclic graph (DAG) with *m* edges can be decomposed into at most *m* weighted paths (Ahuja et al, 1988). Such a decomposition can be computed in polynomial time by iteratively removing weighted paths that saturate at least one edge. However, the optimization version of the problem where we seek an FD with a *minimum* number of paths (minimum flow decomposition [MFD]) is NP-hard (Vatinlen et al, 2008), even on DAGs.

It is also hard to approximate: Hartman et al (2012) showed that there is some *ɛ* > 0 such that MFD cannot be approximated to within a (1 + *ɛ*) factor, unless *P* = NP. The current best approximation ratio for the problem is exponential and was given by Mumey et al (2015). More recent work by Kloster et al (2018) showed that the problem is fixed-parameter tractable (FPT), where the parameter is the size of the minimum decomposition. It is also possible to decompose all but an *ɛ*-fraction of the flow within a *O*(1/*ɛ*) factor of the optimal number of paths (Hartman et al, 2012). On the heuristic side, approaches have centered on greedy methods that choose the widest or longest paths (Vatinlen et al, 2008) in the network. Shao and Kingsford (2017a) showed that these methods can be improved by making iterative modifications to the flow graph before finding a greedy decomposition.

FD is also a key step in numerous applications. For example, some network routing problems (Cohen et al, 2014; Hartman et al, 2012; Hong et al, 2013; Mumey et al, 2015) and transportation problems (Ohst, 2015; Olsen et al, 2020) require FDs that are optimal with respect to various measures. MFDs in particular are used to reconstruct biological sequences such as RNA transcripts [e.g., in tools such as StringTie (Pertea et al, 2015), Scallop (Shao and Kingsford, 2017a), Traph (Tomescu et al, 2013), Ryuto (Gatter and Stadler, 2019), and FlipFlop (Bernard et al, 2014)] and viral quasispecies genomes (e.g., in the tool VG-Flow; Baaijens et al, 2020). However, despite the history of algorithmic work on MFD detailed previously, an exact solver that is fast for instances with large optimal solutions or large flow values has remained elusive. Thus, all practical bioinformatics tools in fact use heuristics for MFD or solve a simpler version of the problem ignoring some information that is available from the sequencing process, resulting in tools that may not reconstruct the correct sequence, even if no other errors are present.

Indeed, various researchers have noted this tradeoff between solving algorithmic problems in DNA assembly exactly and solving them quickly. Nagarajan and Pop (2013) explained that the lack of exact solvers for many of the subproblems involved in DNA sequencing has led to heuristic and ad hoc tools with no provable guarantees on the quality of solutions. In addition, some authors (Bernard et al, 2014; Canzar et al, 2016) have noted that there is a tradeoff between the complexity of the model for RNA assembly (i.e., how much of the true possible solution space that it supports) and its tractability. But if a fast exact solver for MFD exists, this tradeoff may not be necessary for multiassembly.

### 1.1. MFD in multiassembly

One of the most prominent research areas in bioinformatics is the assembly of genetic sequences from short substrings called *reads*, which can be generated cheaply and accurately from next-generation sequencers. In some cases, such as assembly RNA transcripts or viral quasispecies genomes, we must assemble not just a single sequence but a mixed sample of sequences. This version of assembly is called *multiassembly* (Xing et al, 2004). Additional details are provided on both RNA transcript assembly and viral quasispecies genome assembly hereunder.

One mechanism by which complex organisms create a vast array of proteins is alternative splicing of gene sequences, where multiple different RNA transcripts (which are then translated into different proteins) can be created from the same gene (Stamm et al, 2005). In humans, >90% of genes are believed to produce multiple transcripts (Wang et al, 2008). Reconstructing the specific RNA transcripts has proved essential in characterizing gene regulation and function, and in studying development and diseases, including cancer (Kim et al, 2008; Shah et al, 2012). A second multiassembly problem is the reconstruction of viral quasispecies, for example, the different HIV or hepatitis strains present in a single patient sequencing sample, or the different SARS-CoV-2 strains present in a sewage water sample. Because viruses evolve quickly, there can be many distinct strains present at one time, and this diversity can be an important factor in the success or effect of the virus (Vignuzzi et al, 2006).

Although the biological realities underlying the different multiassembly problems may yield some differences in how the problems can be solved, at their heart many approaches contain the algorithmic step of decomposing a network flow into weighted paths. The basic setup and approach for multiassembly is as follows: Given a sample of unknown sequences, each with some unknown abundance (e.g., a set of RNA transcripts or virus strains), all sequences are multiplied and then broken into fragments that can be read by next-generation sequencers to produce millions of sequence reads ranging from hundreds to tens of thousands DNA characters in length.

Many approaches are reference based [e.g., those of Tomescu et al (2013); Trapnell et al (2010); Maretty et al (2014); Pertea et al (2015); Kovaka et al (2019); Bernard et al (2014); Li et al (2011b) for RNA assembly and Zagordi et al (2011); Töpfer et al (2013) for viral quasispecies assembly], meaning that they use a previously constructed reference genome to guide the assembly process. These approaches construct a graph using the sequences contained in the reads where nodes are strings, edges represent overlaps, and weights on edges give the counts of reads that support each overlap. Because a reference is used, these graphs are always DAGs. In the nonreference case (called de novo), graphs may have cycles; we address this further at the end of the article. If errors are minimal, the weights on the edges should form a flow on the network, and the underlying sequences and their abundances must be some decomposition of the flow into weighted paths.

For RNA assembly, recent works by Kloster et al (2018) and Williams et al (2021) have confirmed the common assertion (e.g., by Tomescu et al, 2013; Shao and Kingsford, 2017a; Kovaka et al, 2019; Mao et al, 2020; Zhao et al, 2021; Lin et al, 2012; Mangul et al, 2012) that the true transcripts and abundances should be MFD. No such study has been carried out for viral quasispecies assembly, but existing tools do explicitly seek minimum-sized decompositions (e.g., as in Baaijens et al, 2020; Westbrooks et al, 2008). However, although the abovementioned tools seek minimum-sized FDs, because MFD is NP-hard, they in fact compute decompositions that are not guaranteed to be minimum (and thus may not give the correct assembly, even when no other errors are present).

### 1.2. Limitations of current integer linear programming solutions

One promising direction for fast exact solvers for MFD is integer linear programming (ILP). Existing ILP solvers like Gurobi (Gurobi Optimization, LLC, 2021) and CPLEX (Studio, 2017) incorporate optimizations that allow for fast runtimes in practice for problems that should be hard in general; in fact, ILP is already used in various bioinformatics applications, such as those described in Gusfield (2019). In particular, many existing multiassembly tools use ILP to solve MFD as one step in their process. The basic idea behind these existing formulations is to consider some set of source-to-sink paths through the graph and assign each a binary variable indicating whether or not it is selected in the optimal solution, along with constraints to fully encode the FD problem (i.e., that the selected set of paths—with the weights derived for them by the ILP—form an FD) and to model further practical aspects of the specific multiassembly problem.

However, the number of paths in a DAG is exponential, meaning that if the tools enumerate all paths (and thus can be guaranteed to find the true optimal solution), they are impractical for larger instances. One such example is Toboggan (Kloster et al, 2018), which implements an FPT algorithm for MFD by generating all possible paths. The most common strategy in practical tools is to preselect some set of paths, either for all instances [e.g., VG-flow (Baaijens et al, 2020); CLIIQ (Lin et al, 2012)], or only when the input is large [e.g., MultiTrans (Zhao et al, 2021) and SSP (Safikhani et al, 2013)]. But by preselecting paths, these formulations may not find the optimal MFD solution for the instance.

Although the conference version of this article was in print, the recent RNA transcript assembly method JUMPER (Sashittal et al, 2021) was brought to our attention. JUMPER appears to be, to our knowledge, the only prior method incorporating the search for paths in a DAG into an ILP. However, their solution is slightly less general, because it works only for DAGs having a Hamiltonian path. If Hamiltonicity holds, any source-to-sink path can be encoded as a subset of edges that do not pairwise overlap in the Hamiltonian path (i.e., the tail of an edge does not appear before the head of another edge in the Hamiltonian path). As such, to avoid such pairwise edge overlaps they require a number of constraints that is quadratic in the size of the graph.

### 1.3. Our contributions

We give a new ILP approach to the MFD problem on DAGs, and we show that it can be used on both simulated and real RNA assembly graphs under conditions used in many reference-based multiassembly tools.

In Section 3.1, we show for the first time that it is not necessary to enumerate all paths through a general DAG to encode them in an ILP. The key idea is that any path must have a conserved (unit) flow from its start to its end, and that this concept can be encoded using only a number of variables and constraints that is linear in the size of the graph (rather than exponential, as is the case when the model enumerates all possible paths). This is a standard integer programming method for expressing paths in DAGs, used for example in Taccari (2016).

An implementation of our ILP formulation using CPLEX finds optimal FD solutions on RNA assembly graphs (simulated and assembled from real reads) in <13 seconds over all the datasets tested. This is several times faster than the state-of-the-art MFD solver Toboggan (Kloster et al, 2018), depending on the dataset. Although heuristic solvers such as Catfish (Shao and Kingsford, 2017b) or CoasterHeuristic (Williams et al, 2021) finish within a few seconds, we show that they do not provide optimum solutions. Another benefit of our ILP solutions is that *all* optimum solutions can be reported by the ILP solver, thus potentially helping in “identifying” the correct RNA multiassembly solution, a practical issue acknowledged by both Zheng et al (2022) and Khan et al (2022).

In Sections 3.2 and 3.3, we show that our ILP formulation can be extended to handle common variants on MFD that are solved in practical multiassembly approaches. For example, many tools account for paired-end reads by requiring that they be included in the same path. Another common strategy is to incorporate longer reads such as subpath constraints or phasing paths (Pertea et al, 2015; Shao and Kingsford, 2017a; Williams et al, 2021), which again must be covered by some predicted transcript (i.e., path in an FD). In Section 3.2, we give additional constraints that are expressive enough to not only encode paired-end reads and subpath constraints, but also any generic set of edges that must be covered by a single path [e.g., as when modeling the recent Smart-seq3 protocol producing RNA multi-end reads (Hagemann-Jensen et al, 2020)].

In addition, owing to sequencing or read mapping errors, the weights on edges may not be a flow (i.e., flow conservation might not hold). One approach in this case is to consider intervals of edge weights instead, as in Safikhani et al (2013) and Williams et al (2019). We give a formulation to handle this approach in Section 3.3. Our implementation solves subpath constraint instances in similar time to standard instances, whereas the existing exact solver could not complete on many instances in <60 seconds. Moreover, although the existing interval heuristic is fast, it finds decompositions that are far from optimum. Although all these additional constraints are naturally expressed in ILP (further underlining the flexibility of our approach), the novelty here is their integration with the ILP encoding of all possible paths in the DAG from Section 3.1.

In Section 3.4, we give MFD formulations dealing with the total error over all edges. We can consider an upper bound on the total error, or seek a minimum decomposition that also achieves the minimum error, as studied in Tomescu et al (2015) and used in RNA assemblers such as those given by Li et al (2011a), Li et al (2011b), Bernard et al, (2014), and Tomescu et al (2013). Finally, we note that our formulation could also be used to find decompositions for any of the above variants using a fixed, or upper bounded, number of paths, which is useful if further information is available to restrict the solutions that should be considered.

## 2. PRELIMINARIES

Given a graph *G* = (*V*, *E*), with vertex set *V* and edge set E⊆V×V, we say that s∈V is a *source* if *s* has no in-coming edges. Analogously, we say that t∈V is a *sink* if *t* has no out-going edges. Moreover, we say that *G* is a *DAG* if *G* contains no directed cycles.

**Definition 1** (Flow network). *A tuple G =* (*V*, *E*, *f*) *is said to be a flow network if* (*V*, *E*) *is a DAG with unique source* s *and unique sink t, where for every edge* (u,v)∈E *we have an associated positive integer flow value f_uv_, satisfying conservation of flow for every* v∈V∕{s,t}*, namely:*

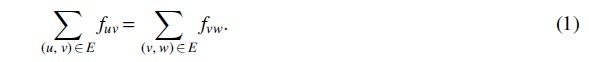

**FIG. 1.**
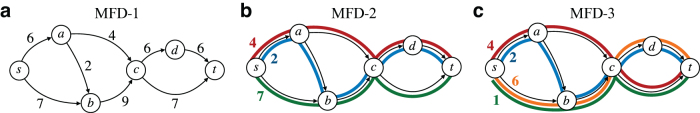
Example of a flow network and of two FDs of it. **(a)** A flow network. **(b)** A 3-flow decomposition into paths of weights (4, 2, 7). **(c)** A 4-flow decomposition into paths of weights (4, 2, 6, 1). FD, flow decomposition.

Given a flow network, a *FD* for it consists of a set of source-to-sink *flow paths*, and associated weights strictly >0, such that the flow value of each edge equals the sum of the weights of the paths passing through that edge. In other words, the superposition of the weighted paths of the FD equals the flow of the network (Fig. 1). Formally:

**Definition 2** *(k-FD)*. *A k-FD* (P,w) *for a flow network G* = (*V*, *E, f*) *is a set of k s−t flow paths* $P=\left(P_{1},\ldots ,P_{k}\right)$ *and associated weights* $w=\left(w_{1},\ldots ,w_{k}\right)$*, with each* $w_{i}\in Z^{+}$*, such that for each edge* (u,v)∈E *it holds that:*

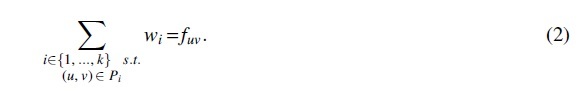

Our above definitions assume integer flow values in the network and integer weights of the flow paths, as is natural because these values count the number of sequenced reads traversing the edges, and are also consistent with previous works such as Kloster et al (2018). However, in practical applications, one could have both fractional flow values and flow path weights, as in for example, Pertea et al (2015). Note also that the integer and fractional decompositions to the problem may differ. For example, Vatinlen et al (2008) observes that there are integer flow networks which admit a *k*-FD with fractional weights, but no *k*′-FD with integer weights, for any k′≤k.

## 3. ILP FORMULATIONS

### 3.1. Minimum flow decomposition

In this section we consider the following problem of finding a minimum size FD.

**Problem 1** (MFD). *Given a flow network G* = (*V*, *E, f*)*, the MFD problem is to find a FD* (P,w) *such that* |P| *is minimized.*

Our solution for problem MFD is based on an ILP formulation of a FD with a given number *k* of paths (a *k*-FD). Using this, one can easily solve the MFD problem by finding smallest *k* such that the flow network admits a *k*-FD. Notice that any DAG admits an FD of size at most |E|, see for example, Ahuja et al (1988) (because one can iteratively take the edge with smallest flow value and create an *s* – *t* path of weight equaling this flow value). Moreover, if assuming integer weights, another trivial upper bound on the size of any FD is |f|, namely the flow exiting *s*, and there is always an FD with |f| paths of weight one.

Thus, if there is a *k*-FD, there is also a *k′*-FD, for all k<k′≤ min{|E|,|f|} (just duplicate a path of weight greater than one, and move weight one from the old copy to the new one). This shows that when searching for the smaller *k* such that the graph admits a *k*-FD we can either do a linear scan in increasing order, or binary search. Because *k* is usually small in our applications, we just do a linear scan. As mentioned at the end of Section 2, the problem can also be defined as allowing real flow values and/or weights. Our ILP formulation can also handle this variant by just changing the domain of the corresponding variables (in which case we will obtain a Mixed Integer Linear Program [MILP]).^*^

We start by recalling the standard formulation of a path used for example by Taccari (2016) for the shortest path problem. If an *s* – *t* path repeats no edge (which is always the case if the graph is a DAG) then we can interpret it simply as the set of edges belonging to the path. If we assign value 1 for each edge on the path, and value 0 for each edge not on the path, then these binary values correspond to a conceptual flow in the graph (*V*, *E*) (different from the input flow). Moreover, this conceptual flow induced by the (single) path is such that the flow out-going from *s* is 1 and the flow in-coming to *t* is 1. It can be easily checked (cf. e.g., Taccari, 2016) that if the graph is a DAG, then this is a precise characterization of an *s* – *t* path.

**FIG. 2.**
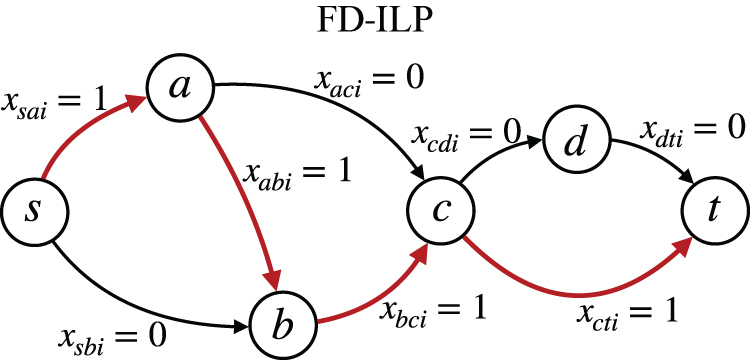
Example of the edge variables of the *i-*th path, satisfying Equations (3a–3c).

Thus, for every path i∈{1,…,k}, and every edge (u,v)∈E, we can introduce a binary variable *x_uvi_* indicating whether the edge (*u*,*v*) belongs to the *i*-th path. The above characterization of a path can be expressed by the following equations (see also Fig. 2):

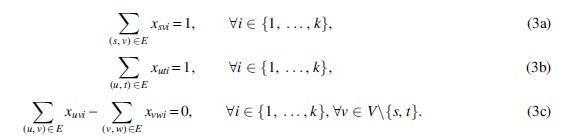

Having expressed a set of *k s* − *t* paths with already known ILP constraints, we need to introduce the new constraints tailored for the *k*-FD problem. That is, we need to state that the superposition of their weights equals the given flow in the network (2). Thus, for each path *i* we introduce a positive integer variable *w_i_* corresponding to its weight, and add the constraint:

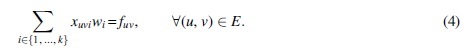

To get the ILP formulation, it remains to linearize Equation (4), which is nonlinear because it involves a product of two decision variables. Let us remark that although nonlinear programming solvers exist [such as IPOPT (Wächter and Biegler, 2006)], they are inefficient, do not scale to a large number of variables, and are nonprofessional grade. Instead, having an ILP formulation means that we can make use of popular solvers such as CPLEX (Studio, 2017) and Gurobi (Bixby, 2007).

Since the decision variables involved in the product in Equation (4) are bounded (*x_uvi_* is binary and *w_i_* is at most the largest flow value of any edge), this equation can be linearized by standard techniques as in for example, Furini and Traversi (2019) and Liberti (2007). For that, we introduce the integer decision variable *π_uvi_*, which represents the product between *w_i_* and *x_uvi_*, and a constant $\bar{w}$ that is a large enough upper bound for any variable *w_i_* (e.g., the largest flow value of any edge). As such, Equation (4) can be replaced by the following equations:

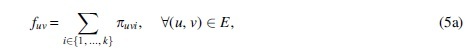

(5b) $$\pi_{uvi}\leq \bar{w}x_{uvi},\forall \left(u,v\right)\in E,\forall i\in \left\{1,\ldots ,k\right\},$$

(5c) $$\pi_{uvi}\leq w_{i},\forall \left(u,v\right)\in E,\forall i\in \left\{1,\ldots ,k\right\},$$

(5d) $$\pi_{uvi}\geq w_{i}-\left(1-x_{uvi}\right)\bar{w},\forall \left(u,v\right)\in E,\forall i\in \left\{1,\ldots ,k\right\}.$$

In these constraints, Equation (5b) ensures that *π_uvi_* is 0 if *π_uvi_* is 0, and Equations (5c) and (5d) ensure that *π_uvi_* is *w_i_* if *x_uvi_* is 1. For completeness, we list hereunder the full ILP formulation for *k*-FD (Table 1).

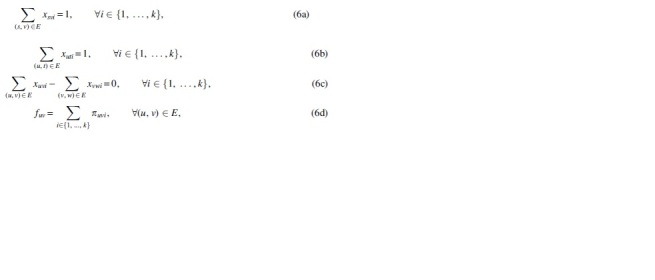

(6e) $$\pi_{uvi}\leq \bar{w}x_{uvi},\forall \left(u,v\right)\in E,\forall i\in \left\{1,\ldots ,k\right\},$$

(6f) $$\pi_{uvi}\leq w_{i},\forall \left(u,v\right)\in E,\forall i\in \left\{1,\ldots ,k\right\},$$

(6g) $$\pi_{uvi}\geq w_{i}-\left(1-x_{uvi}\right)\bar{w},\forall \left(u,v\right)\in E,\forall i\in \left\{1,\ldots ,k\right\},$$

(6h) $$w_{i}\in Z^{+},\forall i\in \left\{1,\ldots ,k\right\},$$

(6i) $$x_{uvi}\in \left\{0,1\right\},\forall \left(u,v\right)\in E,\forall i\in \left\{1,\ldots ,k\right\},$$

(6j) $$\pi_{uvi}\in Z^{+}\cup \left\{0\right\},\forall \left(u,v\right)\in E,\forall i\in \left\{1,\ldots ,k\right\}.$$

**Table 1.** Notation for *k*-Flow Decomposition Integer Linear Programming

Headers	
*x_uvi_*	Binary variable corresponding to the usage of edge (u,v)∈E in flow path i∈{1,…,k}
*w_k_*	Integer variable corresponding to the weight of flow path i∈{1,…,k}
*π_uvi_*	Integer variable corresponding to the product of the weight of flow path i∈{1,…,k} and the usage of edge (u,v)∈E in the same flow path
$\bar{w}$	Sufficiently large upper bound for any *w_i_*, for all i∈{1,…,k}

### 3.2. Subpath constraints

In this section we consider the FD variant where we are also given a set of *subpath constraints* that must appear (as a subpath of some path) in any FD. Among all such decompositions we must find of one with the minimum number of paths. In multiassembly, subpath constraints represent longer reads that span three or more vertices; they are used in popular RNA assembly tools such as StringTie (Kovaka et al, 2019) and Scallop (Shao and Kingsford, 2017a) and their usefulness for that problem was confirmed empirically in Williams et al (2021). Such subpath constraints can also naturally model long RNA-seq reads, and we note that, as several authors also acknowledge [Zhang et al (2021); Amarasinghe et al (2020); Voshall and Moriyama (2018)], long reads do not render the RNA assembly problem obsolete, because they do not always capture full-length transcripts (owing to the conversion from RNA to cDNA), and do not fully capture low-expressed transcripts.

**FIG. 3.**
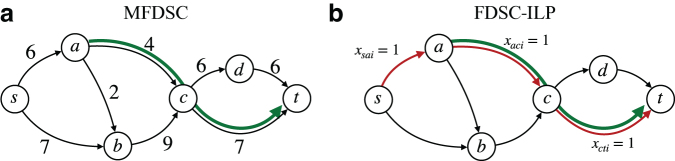
The flow network from Figure 1 with a subpath constraint (which is satisfied by the 4-FD from Fig. 1c, but not by the one in Fig. 1b), and example of a path satisfying the constraint. **(a)** A flow network with a single subpath constraint *R*_1_ = (*a*, *c*, *t*). **(b)** Constraint *R*_1_ is satisfied because for the *i-*th path we can set *r*_i1_ = 1 [and satisfy Eq. (8b)] so that *x_aci_* + *x_cti_* ≥ 2*r_i1_* holds [and satisfy Eq. (8a)].

Formally, the problem can be defined as follows (see also Fig. 3a).

**Definition 3** (FD with subpath constraints). *Let G =* (*V*, *E*, *f*) *be a flow network. Subpath constraints are defined to be a set of simple paths* $ℛ=\left\{R_{1},\ldots ,R_{\ell }\right\}$ *in G (not necessarily s – t paths). FD* (P,w) *satisfies the subpath constraints if and only if*

(7) $$\forall R_{j}\in ℛ,\exists P_{i}\in PsuchthatR_{j}isasubpathofP_{i}.$$

**Problem 2** (Minimum flow decomposition with subpath constraints [MFDSC]). *Given a flow network G =* (*V*, *E*, *f*) *and subpath constraints* ℛ*, the MFDSC problem is to determine if there exists, and if so, find an FD* (P,w) *satisfying [Equation (7)] such that* |P| *is minimized.*

We can expand the previous ILP formulation for *k*-FD to incorporate the conditions necessary to represent the subpath constraints. Let ℛ be the set of simple paths that are required to be part of at least one path of the FD. For each $R_{j}\in ℛ$, we introduce an additional binary variable *r_ij_* denoting the presence of the subpath *R_j_* in the *i-*th path. It clearly holds that *r_ij_* = 1 if and only if for each edge (*u*,*v*) in *R_j_* we have that *x_uvi_ =* 1. Let $\left|R_{j}\right|$ denote the length (i.e., number of edges) of subpath constraint *R_j_*, which is a parameter (i.e., constant). The following inequalities guarantee that each subpath constraint is satisfied by the FD (Fig. 3b):

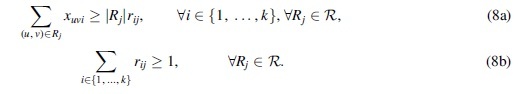

**Remark 1.** *In the above ILP formulation we do not use the fact that the edges of subpath constraint R_j_ are consecutive (i.e., form a path). Thus, the same formulation applies also if the constraint consists of a* pair *of edge-disjoint paths that must all occur in the same transcript, modeling paired-end Illumina reads, or if it consists of a* set *of edge-disjoint paths (or simply of a set of edges), modeling multi-end Smart-seq3 RNA reads (Hagemann-Jensen et al, 2020). More specifically, Equation* (8a) *simply characterizes when all edges of constraint R_j_ are covered by some flow path i, and Equation* (8b) *requires that at least one flow path satisfies the constraint R_j_.*

**Remark 2.** *While for MFD we could modify the ILP to allow also real positive path weights by setting their lower bound to be 0 (because we solve MFD by increasing k, as discussed at the beginning of Section 3.1), this is no longer possible here, since the resulting model could allow as feasible optimum solution a set of k paths decomposing the flow, plus one 0-weight path added just to satisfy some subpath constraints.*

### 3.3. Inexact flow

Another variant of the FD problem is when the given values on the edges of the flow network do not satisfy the conservation of flow property. Instead, they are required to belong to a given interval, for each edge. Thus, we are looking for an *inexact FD*, namely one such that the superposition of its weights belongs to the given interval of each edge. This model was studied in Williams et al (2019) and is used in the practical RNA assembler SSP (Safikhani et al, 2013), which seeks a set of transcripts explaining the read coverage within some user-defined error tolerance (i.e., interval around the observed weights) on all edges.

The problem is formally stated as follows.

**Definition 4** (Inexact flow network). *A tuple* $G=\left(V,E,\underline{f},\bar{f}\right)$ *is said to be an inexact flow network if* (*V,E*) *is a DAG with unique source s and unique sink t, where for every edge* (u,v)∈E *we have associated two positive integer values* $\underline{f_{uv}}$ *and* $\bar{f_{uv}}$*, satisfying* $\underline{f_{uv}}\leq \bar{f_{uv}}$.

**Problem 3** (Minimum inexact flow decomposition [MIFD]; Williams et al, 2019). *Given an inexact flow network* $G=\left(V,E,\underline{f},\bar{f}\right)$ *the MIFD problem is to determine if there exists, and if so, find a minimum-size set of s – t paths* $P=\left(P_{1},\ldots ,P_{k}\right)$ *and associated weights* $w=\left(w_{1},\ldots ,w_{k}\right)$ *with* $w_{i}\in Z^{+}$ *such that for each edge* (u,v)∈E *it holds that:*

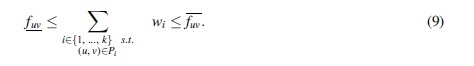

In this variant, the same formulation as presented *k*-FD can be expanded to accommodate the inexact flow component. By simply replacing the flow conservation expressed in Equation (4) [in the linearized form in Eq. (5a)], with the following two constraints:

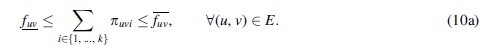

**Remark 3.** *Notice that Equation* (10a) *can be combined with Equations* (8a) *and* (8b) *to obtain a solution if one needs to solve an inexact FD with subpath constraints problem, further underscoring the versatility of the ILP solution in handling various practical variants of the FD problem.*

### 3.4. Imperfect flow

An alternative approach to handle a graph whose weights to not satisfy the flow conservation property flow consists of directly taking the observed read coverages, and trying to find a set of path whose superposition best explains the observed coverages under some error model, penalizing the difference between the observed coverage of an edge and the sum of the weights of the paths going through that edge. This problem has been formalized in Tomescu et al (2015) and also proven NP-hard. To formalize this problem, we denote by *imperfect flow network* any DAG (*V*, *E*) with unique source *s* and unique sink *t*, where for every edge we have an associated integer positive value *f_uv_* (not necessarily satisfying the flow conservation property).

A first formulation of such an MFD variant imposes a fixed bound on the total error of all of the edges.

**Problem 4** (Minimum imperfect FD [bounded error]). *Given an imperfect flow network G =* (*V*, *E*, *f*)*, and an error bound* B≥0*, find (if it exists) a minimum-sized set of s –t paths* $P=\left(P_{1},\ldots ,P_{k}\right)$ *and associated weights* $w=\left(w_{1},\ldots ,w_{k}\right)$ *with* $w_{i}\in Z^{+}$ *such that for each edge* (u,v)∈E

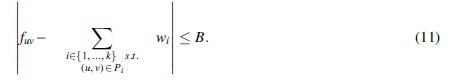

Notice that Problem 4 is a strict generalization of the MFD problem, which is obtained by taking *B =* 0. As carried out in Section 3.3, we can obtain an ILP for it by extending the ILP formulation for *k*-FD to express Equation (11) by the following two sets of linear equations:

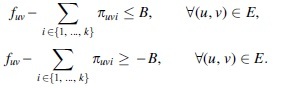

This model is for a fixed *k* value, and a full solution for Problem 4 is obtained by trying all values of *k* in increasing order until the ILP formulation admits a solution. Notice that the same upper bound k≤|E|, because any solution to Problem 4 (i.e., any set of weighted s−t paths) induces a flow, which is decomposable into at most |E| weighted paths.

Another formulation, defined by Tomescu et al (2015) and at the core of RNA multiassembly tools such as Li et al (2011a), Li et al (2011b), Bernard et al (2014), and Tomescu et al (2013), asks to minimize the total sum of squared errors with a minimum number of paths.

**Problem 5** (Minimum imperfect FD [minimum total error]; Tomescu et al, 2015). *Given an imperfect flow network G =* (*V*, *E*, *f*)*, find a set of s – t paths* $P=\left(P_{1},\ldots ,P_{k}\right)$ *and associated weights* $w=\left(w_{1},\ldots ,w_{k}\right)$*, minimizing*

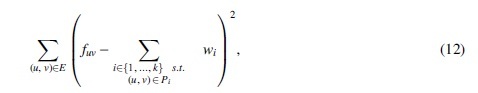

*and among all such sets of paths, find one with minimum k (i.e., with minimum cardinality).*

For a given number *k* of path, Equation (12) can be used as an objective function in an Integer Quadratic Problem, which can solved by commercial solvers such as CPLEX and Gurobi. The main requirement is that the objective function is quadratic and convex, such as:

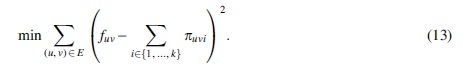

As before, to fully solve Problem 5, one can iterate over *k* from 1 to |E| (upper bound holding by the same reasoning as given previously), and choose the smallest one attaining Equation (13).

## 4. EXPERIMENTS

### 4.1. Experiment design

#### 4.1.1. Solvers

We denote by StandardILP, SubpathConstraintsILP, and InexactFlowILP our ILP formulations for Problems 1 (MFD), 2 (MFDSC), and 3 (MIFD), respectively. We implemented these using the Cplex Python interface under default settings. Our implementations are freely available at github.com/algbio/MFD-ILP. We compare StandardILP with Toboggan, the implementation by Kloster et al (2018) for their exact FPT algorithm for MFD, and with Catfish, the implementation by Shao and Kingsford (2017b) of their heuristic algorithm for MFD. We compare SubpathConstraintsILP with Coaster, the implementation by Williams et al (2021) for MFDSC, which is an exact FPT algorithm extending Toboggan, and also with CoasterHeuristic, which is a heuristic for MFDSC also by Williams et al (2021). We compare InexactFlowILP with IFDSolver, which is an implementation of a heuristic algorithm for MIFD by Williams et al (2019). Given the size of the datasets, we set a time limit for each graph, as also carried out by Kloster et al (2018) and Williams et al (2021) (we use 1 minute in all cases, except that we also include a run of Toboggan with a 5-minute time limit). The runtimes of our ILP implementations include the linear scan in increasing order to find the smallest *k* for which there is a *k*-FD.

#### 4.1.2. Datasets

To test the performance of the solvers under a range of biologically occurring graph topologies and flows weights, we used three human transcriptomic datasets containing a perfect (i.e., the edge weights satisfy conservation of flow) splice graph for each gene of the human genome.

The first dataset, produced by the authors of Shao and Kingsford (2017a) and also used in a number of FD benchmarking studies (Kloster et al, 2018; Williams et al, 2021), was built using publicly available RNA transcripts from the Sequence Read Archive with quantification using the tool (Salmon Patro et al, 2015). We use one of the larger transcriptomes^†^ and call this dataset **SRR020730-Salmon**. We also produce perfect splice graphs by running HiSat2 (Kim et al, 2019) with the provided GRCh38 reference index and then popular RNA assembly tool StringTie (Kovaka et al, 2019) on real RNA reads from SRR307903, and superimposing the resulting transcripts and abundances (after rounding abundances to the nearest integer). We call this dataset **SRR307903-StringTie**. Finally, we create another dataset by directly simulating expression values for all reference transcripts of all genes in the reference genome GRCh.104 *Homo sapiens* by sampling weights from the lognormal distribution with mean −4 and variance 4, as in the default setting of the RNASeqReadSimulator tool (Li, 2014). We multiply the simulated values by 1000 and round to the nearest integer. We call this dataset **Reference-Sim**. For both the **Reference-Sim** and **SRR307903-StringTie** datasets, we use only genes on the positive strand.

For the subpath constraint experiments, we simulate four subpath constraints in each graph as in Williams et al (2021). For four of the groundtruth paths, we take the prefix of the path that includes three nontrivial junctions [equivalent to three edges in the contracted graph described in Kloster et al (2018), Lemma 13] as a subpath constraint. If a splice graph has fewer than four groundtruth paths, it is excluded from this experiment.

For the inexact flow experiments, we simulate interval flows as follows, similar to what was performed in Williams et al (2019). For each true edge flow *f_uv_*, we independently sample a perturbed flow $f\prime_{uv}$ from 
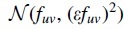
, the Gaussian distribution with mean *f_uv_* and standard deviation *ɛ f_uv_*. For this experiment we fixed *ɛ* = 0.05. We then create intervals as $\left[0.9f\prime_{uv},1.1f\prime_{uv}\right]$ with values rounded to the nearest integer, corresponding to a 10% error tolerance from the observed values. As described in Williams et al (2019), it is possible that an inexact FD instance created in this way is infeasible; if an infeasible instance is created, we re-create it until a feasible instance is found.

From all datasets, the trivial graphs made up of a single path (i.e., admitting a trivial FD) are excluded.

#### 4.1.3. Metrics

For each dataset and each FD variant, we report **min** *k*, the number of paths in an MFD for each problem variant; **Amount**, namely the number of graphs having that specific value of **min** *k*; **Avg.**, the average time (in seconds) for each instance solved within the time limit; **Σ**, the total time (in seconds) required to solve all instances (this included also the running time of the instances that did not finish within the time limit); **Solved**, the percentage all instances solved within the time limit; **Diff.**, the average difference between the number of paths obtained with a heuristic algorithm and the optimum one.

### 4.2. Results

The results for Problem MFD are given in Table 2. For all three datasets, the average time and the total time of Toboggan and Catfish outperform StandardILP for less complex genes, where the number of flowpaths is at most 10 or 15. However, as the genes becomes more complex (larger optimum FDs), StandardILP is capable of solving all instances within an average of 10 seconds, whereas Toboggan and Catfish require on average 16 and 11 seconds for the solved instances, respectively. In addition, Toboggan does not solve all instances even within the 5-minute time limit. Recall also that Catfish is a heuristic, and thus it does not always return optimum solutions (see column **Diff.**).

**Table 2.** Results for Problem Minimum Flow Decomposition

	Min *k*	Amount	StandardILP			Toboggan (1 minute)			Toboggan (5 minutes)			Catfish			
			Avg.	Σ	Solved	Avg.	Σ	Solved	Avg.	Σ	Solved	Avg.	Σ	Solved	Diff.
SRR020730 Salmon	2–5	34371	0.091	3127	100	0.002	68	100	0.002	68	100	0.001	34	100	0.00
	6–10	2291	0.204	467	100	0.023	52	100	0.024	54	100	0.031	71	100	0.00
	11–15	95	4.692	445	100	2.361	225	100	2.612	248	100	3.582	340	100	2.85
	16–20	16	5.891	94	100	10.453	287	86	22.531	671	93	8.451	135	100	3.75
	21–max	7	10.222	71	100	16.564	281	50	33.221	643	78	11.621	81	100	4.56
Reference Sim	2–5	14513	0.089	1303	100	0.002	29	100	0.003	43	100	0.058	841	100	0.00
	6–10	1506	0.352	530	100	0.124	186	100	0.123	186	100	0.124	186	100	0.00
	11–15	261	4.564	1191	100	24.132	4365	75	29.312	6575	92	1.299	339	100	2.79
	16–20	63	10.332	650	100	36.344	1753	65	46.444	3759	83	10.45	658	100	3.75
	21–max	41	12.833	526	100	54.732	1553	51	57.672	4268	73	31.65	1298	100	4.56
SRR30790 StringTie	2–5	7335	0.122	894	100	0.022	161	100	0.022	162	100	0.029	212	100	0.00
	6–10	768	1.051	807	100	1.191	914	100	1.191	915	100	0.172	132	100	0.00
	11–15	133	4.855	645	100	5.063	2535	71	10.343	5998	88	3.871	514	100	2.53
	16–20	55	6.895	378	100	12.451	1764	57	21.561	5167	74	5.452	299	100	3.75
	21–max	37	10.512	388	100	20.562	1433	51	32.211	4362	68	9.651	357	100	4.56

Among the different datasets, **SRR020730-Salmon** has fewer complex genes and most instances are solved more easily. However for **SRR307903-StringTie** (constructed from real RNA reads) and **Reference-Sim** datasets, there is a larger amount of complex genes and consequently fewer instances can be solved by Toboggan and Catfish, whereas StandardILP remains efficient and scalable. In these results, although StandardILP does not perform as fast as on **SRR020730-Salmon**, its runtime is still competitive, it can be scaled to graphs with larger *k* without compromising its efficiency. On the contrary, Toboggan's runtime is exponential in the size of the optimum decomposition, which hinders its usage on larger instances. Moreover, notice that in some applications (e.g., cancer transcriptomics; Huang et al, 2021) the graphs of interest do have a large number of RNA transcripts because of the genetic mechanism driving the disease. Hence, in such applications the need to find a FD is even greater for large *k*.

Finally, one of the key steps in the Toboggan implementation is a reduction of the graph [to simplify nodes with in-degree *or* out-degree equal to one, see Kloster et al (2018)], which is a key insight behind its efficiency. However, this observation is highly tailored to the MFD problem, and cannot be easily extended to other FD variants (in fact, it is not used by real RNA assemblers).

The results for Problem MFDSC are given in Table 3. For all three datasets, SubpathConstraintsILP is capable of solving instances of any size within a few seconds. As an ILP formulation, the addition of the constraints corresponding to the subpath constraints does not hinder its scalability or efficiency. On the contrary, Coaster is both slow on small instances, and does not solve large instances. This shows that the Toboggan implementation is optimized to use many properties of the standard MFD problem, that are not generalizable to variants of it of practical applicability, such as Problem MFDSC. Moreover, similar to the Catfish heuristic, CoasterHeuristic does not return optimum solutions.

**Table 3.** Results for Problem Minimum flow Decomposition with Subpath Constraints

	Min *k*	Amount	SubpathConstraintsILP			Coaster			CoasterHeuristic			
			Avg.	Σ	Solved	Avg.	Σ	Solved	Avg.	Σ	Solved	Diff.
SRR020730 Salmon	4–10	5691	0.192	1082	100	30.123	176823	85	0.005	28.5	100	2.14
	11–15	95	1.475	139	100	45.121	4367	44	0.014	1.33	100	3.04
	16–20	16	3.461	55	100	60.000	960	0	0.025	0.04	100	3.91
	21–max	8	10.452	83	100	60.000	480	0	0.067	0.536	100	4.51
Reference Sim	4–10	6512	0.18	1167	100	37.132	243963	84	0.006	39.1	100	3.13
	11–15	260	1.10	279	100	46.211	12097	14	0.031	1.12	100	4.12
	16–20	78	2.58	203	100	60.000	4680	0	0.041	0.32	100	5.12
	21–max	40	11.51	460	100	60.000	3000	0	0.064	2.54	100	8.13
SRR30790 StringTie	4–10	864	0.181	329	100	28.241	244001	86	0.006	5.18	100	2.98
	11–15	104	1.124	148	100	45.142	4693	25	0.032	0.32	100	3.07
	16–20	70	2.578	250	100	60.000	4200	0	0.083	0.58	100	4.14
	21–max	27	11.51	391	100	60.000	1620	0	0.091	2.42	100	5.78

The results for Problem MIFD are given in Table 4. For all three datasets, both formulations run on any instance in a small amount of time. In fact, InexactFlowILP generally has the same running time as StandardILP, which further underscores the flexibility and efficiency of our formulations. However, IFDSolver is a heuristic solver, having a significant difference with respect to the size of a minimum decomposition even for small *k*.

**Table 4.** Results for Problem Minimum Inexact Flow Decomposition

	Min *k*	Amount	InexactFlowILP			IFDSolver			
			Avg.	Σ	Solved	Avg.	Σ	Solved	Diff.
RR020730 Salmon	2–5	34371	0.087	2990	100	0.001	34	100	2.12
	6–10	2291	0.131	300	100	0.025	57	100	2.41
	11–15	95	4.784	454	100	0.134	12	100	3.51
	16–20	16	5.784	91	100	0.618	10	100	4.13
	21–max	7	10.16	70	100	1.124	8	100	5.17
Reference Sim	2–5	14513	0.153	2165	100	0.003	44	100	2.56
	6–10	1506	0.109	164	100	0.052	78	100	2.78
	11–15	261	3.132	817	100	0.254	66	100	3.64
	16–20	63	5.791	364	100	0.783	50	100	3.34
	21–max	41	11.56	473	100	1.341	55	100	3.56
SRR30790 StringTie	2–5	7335	0.104	762	100	0.001	7	100	2.34
	6–10	768	0.219	168	100	0.047	36	100	2.41
	11–15	133	2.891	384	100	0.345	45	100	3.40
	16–20	55	6.183	340	100	0.871	48	100	3.21
	21–max	37	13.214	488	100	1.091	40	100	3.78

## 5. CONCLUSIONS AND FUTURE WORK

FD is a key problem in computer science, with applications in various fields, including the major multiassembly problems from bioinformatics. Despite this, the only exact solution for MFD is the FPT algorithm of Kloster et al (2018), which does not scale to large values of *k*, and cannot be efficiently extended to model practical features of real data (such as long reads, or inexact flows). In fact, a large number of practical RNA assemblers use an ILP formulation at their core, thanks to their flexibility in modeling various aspects of real data. However, such formulations are based either on an impractical exhaustive enumeration of all possible *s – t* paths, or on a greedy heuristic to select a smaller set of candidate *s – t* paths that might be part of an optimum solution.

In this article we show an efficient quadratic-size ILP for MFD and variants, avoiding for the first time the current limitation of (exhaustively) enumerating candidate *s – t* paths. We also show that many constraints inside state-of-the-art RNA assemblers can be easily modeled on top of our basic ILP (i.e., subpath constraints, inexact, and imperfect flows). Further flexibility also comes from the fact that all our ILPs are based on modeling a specific type of FD with a *given*, or *upper bounded* number *k* of paths (thus, they do not need to solve the minimum version of the problem). On both simulated and real datasets, we show that our ILP formulations finish within 13 seconds on any instance, and within a few seconds on most instances.

On the practical side, we hope that our flexible ILP formulations can lie at the core of future reference-based RNA assemblers employing *exact* solutions. Thus, the current tradeoff between the complexity of the model and its tractability might not be necessary anymore. On the theoretical side, our ILP formulation represents the first *exact* solver for MFD scaling to large values of *k*, and it could be a reference when for example, benchmarking various other heuristic or approximation algorithms.

Given the maturity of ILP solvers and Toboggan's intrinsic exponential dependence on *k*, it is not surprising that an ILP for MFD using a quadratic number of variables performs significantly better than Toboggan for larger *k* values. However, since for small *k* values our ILP formulations are still slower, as future work it would be interesting to further devise more efficient MFD solvers (e.g., as a start, run Toboggan when the instance is detected as being “small enough”).

It would also be interesting to extend our ILP formulations to flow networks with cycles. While in this work we focus on reference-based approaches for multiassembly, de novo approaches [e.g., Grabherr et al (2011); Schulz et al (2012) for RNA assembly and Baaijens et al (2019), Baaijens et al (2020); Posada-Céspedes et al (2021); Chen et al (2018) for viral quasispecies assembly] may yield graphs with cycles. In this context, any flow in such a network can be decomposed into at most |E| weights *s – t* paths and cycles.

## Notes

^*^
We note that this version has one subtlety to address: as discussed below, it is necessary to linearize products in the formulation to make it a true ILP (or MILP, in this case). To linearize products of the *real* variables, it is required that the real variables have closed bounds. However, if we solve *k*-FD for increasing *k* (and not binary search), we can use $w_{i}\geq 0$, since no weight 0 path will be included. This introduces the limitation that this formulation could not be used to solve flow decomposition for a fixed *k*, but only if *k* is an upper bound on the solution size.

^†^
The full dataset from Shao and Kingsford (2017b) is available at (https://zenodo.org/record/1460998). We use the file (rnaseq/sparse_quant_SRR020730.graph).
